# Differences in words used to describe racial and gender groups in Medical Student Performance Evaluations

**DOI:** 10.1371/journal.pone.0181659

**Published:** 2017-08-09

**Authors:** David A. Ross, Dowin Boatright, Marcella Nunez-Smith, Ayana Jordan, Adam Chekroud, Edward Z. Moore

**Affiliations:** 1 Department of Psychiatry, Yale University School of Medicine, New Haven, CT, United States of America; 2 Department of Emergency Medicine, Yale University School of Medicine, New Haven, CT, United States of America; 3 Department of General Internal Medicine, Yale University School of Medicine, New Haven, CT, United States of America; 4 Department of Epidemiology, Yale School of Public Health, New Haven, CT, United States of America; 5 Department of Psychology, Yale University, New Haven, CT, United States of America; 6 Department of Engineering, Central Connecticut State University, New Britain, CT, United States of America; Oregon Health and Science University, UNITED STATES

## Abstract

**Purpose:**

The transition from medical school to residency is a critical step in the careers of physicians. Because of the standardized application process–wherein schools submit summative Medical Student Performance Evaluations (MSPE’s)–it also represents a unique opportunity to assess the possible prevalence of racial and gender disparities, as shown elsewhere in medicine.

**Method:**

The authors conducted textual analysis of MSPE’s from 6,000 US students applying to 16 residency programs at a single institution in 2014–15. They used custom software to extract demographic data and keyword frequency from each MSPE. The main outcome measure was the proportion of applicants described using 24 pre-determined words from four thematic categories (“standout traits”, “ability”, “grindstone habits”, and “compassion”).

**Results:**

The data showed significant differences based on race and gender. White applicants were more likely to be described using “standout” or “ability” keywords (including “exceptional”, “best”, and “outstanding”) while Black applicants were more likely to be described as “competent”. These differences remained significant after controlling for United States Medical Licensing Examination Step 1 scores. Female applicants were more frequently described as “caring”, “compassionate”, and “empathic” or “empathetic”. Women were also more frequently described as “bright” and “organized”.

**Conclusions:**

While the MSPE is intended to reflect an objective, summative assessment of students’ qualifications, these data demonstrate for the first time systematic differences in how candidates are described based on racial/ethnic and gender group membership. Recognizing possible implicit biases and their potential impact is important for faculty who strive to create a more egalitarian medical community.

## Introduction

Ethnic/racial and gender disparities are prevalent in healthcare, including medical education. Historically under-represented minority (hURM) students have been reported to receive lower clerkship grades than White counterparts in medical school [1] and are less likely to be inducted into Alpha Omega Alpha (the premier medical school honor society) [2], and women and hURM’s in academic medicine report a greater experience of discrimination [3–5]. Women and individuals from hURM backgrounds appear to be promoted less frequently at medical schools to the ranks of Associate and Full Professor [6, 7]. Numerous studies have shown that women are described differently than male counterparts in letters of recommendation [8–12].

While there may be many factors contributing to such differences, some variation in how groups advance and in their subjective experience is thought to reflect discrimination by evaluators [13], including unconscious gender-linked [14] and ethnic/racial stereotypes [15]. One way to explore how biases might affect medicine is to look at the transition from medical school into residency training because of the highly standardized process through which all medical school graduates must apply.

The core document in each application is the Medical Student Performance Evaluation (MSPE; colloquially referred to as the “Dean’s Letter”). The relationship between ethnicity/race and gender and the way in which applicants are described in this document has only been minimally explored. The largest study included 297 applications (227 men, and 70 women) and looked only at differences in gender [16]. To the best of our knowledge, there are no studies examining the differences based on race/ethnicity in the MSPE.

Here we report data regarding the use of language in the MSPE’s from 6,000 applicants to residency programs at our host institution in the 2014–2015 application cycle. We hypothesized that female and hURM applicants would be described differently from male and White applicants, respectively, according to well-established social stereotypes.

## Methods

### Study design and population

This was a retrospective cohort study of 6,000 MSPE’s from graduates of 134 US allopathic medical schools submitted to sixteen residency programs at the Yale School of Medicine during the 2014–2015 application cycle. We did not include applications from international or Caribbean medical schools because the hypotheses we sought to test regarding possible implicit biases are socially mediated and may vary considerably based on local culture. We did not include applications from osteopathic medical schools due to differences in the structure of their curriculum, including that students are not required to take the United States Medical Licensing Examinations (USMLE; see discussion in Methods, below). The Yale University Institutional Review Board deemed this study exempt from review (exemption category 4; HIC# 1408014491).

### Study protocol

A literature review was conducted to identify words and themes related to potential implicit biases within letters of recommendation for students or faculty in academic medicine. After extensive review, 24 words from four thematic categories (as previously described in the literature) were selected based on prior results and pertinence to this study [9, 16, 17]. The four categories included adjectives describing standout traits, ability, grindstone habits (i.e. work ethic), and compassion.

We used Matlab (Mathworks) to extract from the MyERAS section of the application each individual’s self-described race and ethnicity, gender, age, and medical school. We then used Matlab to extract from each MSPE the number of occurrences of the individual words within our defined thematic categories. Some adjectives are used by particular schools as an indicator of class rank; in these cases, the MSPE often contains common-text explaining the school’s system. For example, one university writes in the first page of every MSPE: “We provide a grouping of ‘outstanding,’ ‘excellent,’ ‘very good,’ or ‘good,’ which is determined by the student’s performance…” Because of this common text, each student at that school will have each of these adjectives appear at least once in their MSPE in a manner that does not relate to that student’s personal attributes or performance. To account for this effect, for each school the software counted the minimum number of times that each word of interest was used across all applicants and then subtracted those values from each applicant’s word count. For example, if all applicants from a school had the word “excellent” appear at least 5 times, the software subtracted 5 from each applicant’s count of this word.

Some applicants applied to multiple programs (N = 358)–their MSPE was included only once. Due to technical difficulties obtaining applications, data concerning race and ethnicity were not available for applicants applying to pathology, otolaryngology, urology, and plastic surgery. Consequently, MSPE’s from these fields were not included in the analysis on race and ethnicity.

### Statistical analyses

Outcome data from the MSPE analyses were extracted using in-house software and then analyzed using STATA Version 14 (StataCorp. 2015).

Demographic characteristics of applicants were summarized. Chi-squared tests were then performed to analyze for each specified keyword the percent of applicants described at least once using that word by gender and race/ethnicity. A Bonferroni correction was applied to account for multiple comparisons (corrected alpha = 0.002).

A multivariable logistic regression was then performed to model the effect of race/ethnicity on the likelihood of an applicant being described by a particular word in the MSPE after controlling for USMLE Step 1 scores (in order to account for potential group differences that may correlate with test scores).

## Results

The sample of applicants reviewed in the present study was comparable to national data on medical school seniors with respect to applicant gender, race, ethnicity, and age (Table 1) [18, 19]. Applicants analyzed in the study were more likely to be from medical schools ranked in the top 40 by NIH funding and more likely to be inducted into the Alpha Omega Alpha (AOA) honor medical society.

**Table 1:** Characteristics of US medical school seniors nationally and study population.

	US Seniors Nationally	Study Cohort
n =	16,362	6,000
Gender (Female)	47%	45%
Race/Ethnicity*		
Black	5%	7%
Hispanic	5%	4%
Asian	21%	26%
White	58%	55%
USMLE Step 1 (median)	229	237
AOA %	15%	19%
Top 40 Med School (NIH Funding)	33%	36%
Ph.D. %	4%	4%

*n = 5,014 for Race/Ethnicity secondary to exclusions based on race/ethnicity screening in applications and individuals who chose not to respond

The median age of applicants was 26, and 45% of applicants were women. The dataset was 7% Black, 4% Hispanic, 24% Asian, and 55% White. Thirty-six percent of applicants attended a school ranked in the top 40 by NIH funding and the median USMLE Step 1 score was 237. Demographic characteristics of the data set are illustrated in Table 2. Specialty breakdown of applications is shown in Table 3.

**Table 2:** Population demographic characteristics.

** **	Black (n = 346)	Hispanic (n = 202)	White (n = 2,740)	Asian (n = 1,281)	Multi (n = 336)	Other (n = 109)
**Age (median)**	27	26	27	26	27	26
**Gender (Female)**	193 (56%)	101 (50%)	1,217 (44%)	599 (47%)	161 (48%)	45 (41%)
**Gold Humanism**	30 (9%)	24 (12%)	336 (12%)	92 (7%)	28 (8%)	10 (9%)
**USMLE Step 1 (mean)**	221	227	240	236	235	234
*<25th Percentile*	25%	13%	6%	9%	11%	16%
*25th-75th Percentile*	50%	54%	34%	40%	41%	37%
*>75th Percentile*	25%	33%	60%	51%	48%	47%
**Top 40 Med School (NIH Funding)**	117 (34%)	72 (36%)	996 (36%)	489 (38%)	70 (45%)	137 (41%)

**Table 3:** Population specialty breakdown.

Specialty	n =	%
Anesthesia	606	10%
Internal Medicine	1626	27%
Neurology	192	3%
Neurosurgery	205	3%
Obstetrics and Gynecology	363	6%
Orthopedic Surgery	562	9%
Otolaryngology	303	5%
Pathology	161	3%
Pediatrics	629	10%
Plastic Surgery	170	3%
Psychiatry	425	7%
Radiology	302	5%
Surgery	470	8%
Thoracic Surgery	69	1%
Urology	231	4%
Vascular Surgery	44	1%
Total	6,358	106%

n > 6,000 due to 358 individuals who applied to multiple programs

Table 4 shows the percent of applications in which the descriptive word was used at least once in each MSPE by race and ethnicity. A significant difference was found in the use of the standout words “exceptional”, “best”, and, “outstanding” with White applicants being more likely than Blacks, Hispanics, and Asians to be described with these adjectives. Concerning the thematic category of ability, Whites were also statistically more likely to be described as “bright” when compared to Blacks, Hispanics, and Asians. Multivariate regression showed that while USMLE Step 1 scores showed a small but significant correlation with standout keywords, race/ethnicity group differences in the use of these adjectives remained significant after controlling for these scores (see Table 5; all χ^2^(5, N = 5,014) > 15, p< .01).

**Table 4:** Percentage of applicants by race/ethnicity group for whom each descriptive word was used at least once in the Medical Student Performance Evaluation.

** **	Black	Hispanic	White	Asian	Multi	Other	p-value
**Word Categories**	n = 346	n = 202	n = 2,740	n = 1,281	n = 336	n = 109	(* Alpha = .002)
**Standout Words**							
Exceptional	50%	52%	64%	54%	64%	58%	<0.001*
Best	41%	44%	54%	49%	50%	58%	<0.001*
Outstanding	77%	84%	86%	79%	82%	88%	<0.001*
Superb	30%	32%	38%	36%	38%	38%	0.025
Stellar	7%	7%	10%	8%	9%	13%	0.067
Excellent	91%	90%	93%	93%	95%	97%	0.050
Phenomenal	3%	5%	5%	5%	5%	8%	0.213
**Ability**							
Intelligent	40%	43%	49%	50%	46%	44%	0.004
Bright	43%	44%	57%	54%	54%	52%	<0.001*
Talent	19%	18%	20%	19%	17%	15%	0.760
Brilliant	3%	1%	3%	3%	4%	2%	0.420
Competent	40%	20%	29%	27%	32%	34%	<0.001*
Smart	19%	18%	24%	23%	24%	28%	0.129
Gifted	5%	5%	6%	5%	7%	5%	0.342
**Grindstone**							
Organized	71%	74%	80%	77%	82%	79%	0.001*
Hardworking	76%	74%	77%	78%	77%	77%	0.790
Conscientious	36%	28%	32%	34%	33%	37%	0.337
Diligent	42%	32%	36%	37%	34%	31%	0.115
**Compassion**							
Caring	47%	50%	51%	49%	51%	55%	0.750
Kind	35%	32%	33%	34%	36%	42%	0.332
Empathy	36%	49%	40%	35%	38%	45%	0.003
Compassionate	56%	49%	54%	51%	51%	63%	0.480

**Table 5:** Associations between race/ethnicity and adjective use controlling for USMLE Step 1 scores.

	Odds Ratios				
	(95% Confidence Interval)				
Applicant Characteristics	Exceptional	Best	Outstanding	Bright	Competent
Race/ethnicity*					
Black	1 [Reference]	1 [Reference]	1 [Reference]	1 [Reference]	1 [Reference]
White	1.39 (1.09–1.75)	1.38 (1.09–1.75)	1.31 (0.99–1.74)	1.47 (1.16–1.86)	0.62 (0.49–0.79)
Hispanic	0.98 (0.69–1.40)	1.02 (0.71–1.45)	1.41 (0.89–2.22)	0.97 (0.68–1.38)	0.38 (0.26–0.58)
Asian or Pacific Islander	0.94 (0.74–1.20)	1.20 (0.94–1.53)	0.85 (0.64–1.14)	1.39 (1.09–1.77)	0.57 (0.44–0.74)
Multiracial	1.49 (1.09–2.04)	1.25 (0.92–1.70)	1.08 (0.74–1.59)	1.41 (1.04–1.91)	0.72 (0.53–0.99)
Other	1.16 (0.75–1.81)	1.75 (1.12–2.71)	1.88 (0.99–3.56)	1.31 (0.85–2.02)	0.78 (0.49–1.23)
USMLE Step 1 Score	1.02 (1.01–1.02)	1.01 (1.01–1.01)	1.02 (1.02–1.02)	1.01 (1.01–1.01)	0.99 (0.99–1.00)

*As a category, the p-value for Race/ethnicity using a Wald test was <0.01 for each adjective

The adjective “competent” was used more frequently to describe Blacks than any other racial or ethnic group. Use of the word “competent” did not correlate with USMLE Step 1 scores. Based on this finding, a specific contextual analysis for the adjective “competent” was performed. Excerpts of the two sentences before and after each occurrence of the word “competent” were extracted from individual MSPE’s and analyzed by three physicians. These physicians were chosen based on their experience in compiling and reading MSPE’s and were blind to the race and ethnicity of the applicants in the MSPE’s being reviewed. A total of 50 excerpts were reviewed with an oversampling of Black applicants (40% Black, 20% Hispanic, 20% White, 20% Asian). Each reviewer judged whether the use of “competent” had a positive, neutral, or negative connotation.

Contextual analysis by reviewers demonstrated that the adjective “competent” had a positive connotation 37% of the time when describing Blacks compared to 33% for Hispanics, 57% for Whites, and 60% for Asians, *p* = 0.052.

There was a difference at the threshold of significance for use of the word “organized” with White applicants being more likely to be described as “organized”. No statistically significant difference was found in the use of other grindstone words or for words in the category of compassion by ethnicity/race.

Table 6 shows the percent of applications in which each word from the four thematic categories was used at least once in the MSPE by gender. Women were more likely than men to be described as “caring”, “compassionate”, and “empathetic” or having “empathy”. Additionally, women were more likely to be described as “bright” and “organized”. No statistically significant difference was found in the use of standout or grindstone adjectives.

**Table 6:** Descriptive word categories by gender.

	Women	Men	P-value
**Word Categories**	n = 2,554	n = 3,131	* significant <0.002
**Standout Words**			
Exceptional	61%	58%	0.072
Best	52%	50%	0.142
Outstanding	84%	82%	0.012
Superb	38%	35%	0.011
Stellar	10%	8%	0.019
Excellent	93%	92%	0.258
Phenomenal	5%	5%	0.979
**Ability**			
Intelligent	48%	49%	0.734
Bright	58%	52%	<0.001*
Talent	20%	18%	0.22
Brilliant	3%	3%	0.734
Competent	30%	28%	0.04
Smart	23%	22%	0.416
Gifted	6%	5%	0.076
**Grindstone**			
Organized	80%	75%	<0.001*
Hardworking	77%	77%	0.523
Conscientious	34%	31%	0.027
Diligent	37%	35%	0.128
**Compassion**			
Caring	55%	45%	<0.001*
Kind	35%	32%	0.03
Empathy	43%	33%	<0.001*
Compassionate	58%	48%	<0.001*

## Discussion

This study addresses a gap in knowledge concerning how descriptive language used by letter writers in the MSPE differs by gender and race/ethnicity of applicants to residency programs. Previous studies have assessed the impact of gender on letters of recommendation to residency programs; however, most of these studies were limited in sample size. Moreover, none of the previous studies addressed the relation between race/ethnicity on how applicants are described in the MSPE.

Although the American Association of Medical Colleges (AAMC) has established guidelines to standardize the MSPE, significant heterogeneity in the document persists across schools. Some MSPE’s include long and detailed quotes from each clinical rotation while others are terse. Some MSPE’s include extended summative narratives that are written by the Dean (or a representative) while others employ keywords that indicate a summative ranking.

Our data demonstrate that descriptive language used in the MSPE varies by group. In our sample, White applicants were more likely to be described with standout words such as “outstanding”, “exceptional”, and “best” when compared to Blacks, Asians, and Hispanics. Moreover, women were more likely than men to be described with words related to compassion such as “kind”, “caring”, and “empathic”. Interestingly, the ability adjective “competent” was the only descriptor used more frequently for Blacks than any other race or ethnic group. Our additional contextual analysis of the use of “competent” showed that the adjective was less likely to have a positive connotation when describing Blacks and Hispanics. This suggests that the term may be used as a word of minimal assurance when describing Blacks and Hispanics.

A critical question is the extent to which implicit bias by the authors of the MSPE may have contributed to the differences found in our study. Differences in the use of standout and ability words among applicants by race/ethnicity remained significant even after controlling for USMLE Step 1 scores. In an ideal world, one might hope to identify other standardized measures of performance in medical school that could be used as independent variables to study the possible role of implicit biases in the MSPE. The most obvious of these would be clerkship performance. However, for several reasons, this is not currently feasible (including different grade distributions and variable criteria for how grades are determined among medical schools). Analysis of such data would enable exploration of whether there may be group differences in clerkship performance based on race/ethnicity and gender, whether implicit beliefs of supervisors and peers contribute to group differences in performance where there are any, and/or whether implicit beliefs play a role in the subjective components of clinical evaluation even in the absence of differences in performance.

Unconscious bias concerning gender, race, and ethnicity has been well documented across a range of social settings (e.g. job or housing applications), even when other demographic variables and qualifications have been held constant [20]. Our study raises the question of whether implicit bias or even explicit stereotypes may also contribute to how medical students are perceived and described in the MSPE. Recognition of implicit bias and its impact on letters of evaluation is important for medical school Deans when writing MSPE’s and for applicant reviewers as we strive to create a more egalitarian medical community.

Of note, this issue is especially timely as the AAMC recently released a document outlining “Recommendations for Revising the Medical Student Performance Evaluation (MSPE)” [21]. According to this document: “The purpose of the MSPE is not to advocate for the student, but rather to provide an honest and objective summary of the student’s personal attributes, experiences, and academic accomplishments based, to the greatest degree possible, on verifiable information and summative evaluations. When possible, comparative assessments of the student’s attributes, experiences, and accomplishments relative to their institutional peers should be provided…”. The task force creating this document also outlines stated principles (among others) of: enhancing “standardization and transparency that facilitates the residency selection process”; “[increasing] opportunity for program directors to examine applicants holistically in the pre-interview stage”; and including “qualitative and quantitative assessments of applicants in an easy to read format”. The present data emphasize the challenge that Deans face in drafting these documents: in trying to incorporate holistic, narrative descriptions of applicants it is possible that implicit biases may undermine their objectivity.

There are limitations to this study that should be noted. Although our large sample came from 134 medical schools (95% of all US schools), including applications to 16 different residency specialties and reflecting more than 30% of the total number of US applicants, they were all submitted to a single institution (Yale University) during the 2014–2015 academic year. This sample contains an over-representation of schools from the Northeast (39% of our applications; 19% of applications were from the Midwest; 32% from the South; and 10% from the West). We also chose to analyze applications only from allopathic medical schools in the United States. Our conclusions should be considered within the scope of this particular population. Data on race and ethnicity were not available for students applying to otolaryngology, pathology, plastic surgery, and urology.

We would also note that the words selected for the present study were chosen based on a careful review of the extant literature–it is obviously possible that groups may differ based on other words (or bigrams or other combinations). Such possibilities might be explored using a computational linguistics approach that would not rely on *a priori* hypotheses. Nonetheless, the findings from the present study are clearly both significant and important–e.g. there can be no doubt that a Program Director will respond favorably to an applicant being described in the MSPE as the “best”.

The results of our study have implications for future research. First, the current findings show differences in key word use in what is supposed to be the most objective aspect of the application. Additional studies should continue to explore the relationship between how applicants are described in the MSPE and other measures of performance. Relatedly, it will be important to evaluate the effect that medical school characteristics, such as size, geography, faculty diversity, student diversity, and racial climate have on the manner in which students are discussed in the MSPE. Finally, future work should explore how various factors impact residency match outcomes–including number of interviews offered and how programs ultimately rank applicants. In addition to MSPE keywords, this could include language usage in letters of recommendation and other factors, such as applicant photos–all of which have the potential to unfairly influence the selection of applicants into the limited opportunities for graduate medical training.
